# Chlorido[1-(2-eth­oxy­phen­yl)3-(4-nitro­phen­yl)triazenido]mercury(II)

**DOI:** 10.1107/S1600536810027819

**Published:** 2010-07-21

**Authors:** Mohammad Reza Melardi, Zeynab Roohi, Nazanin Heidari, Mohammad Kazem Rofouei

**Affiliations:** a Department of Chemistry, Islamic Azad University, Karaj Branch, Karaj, Iran; b Faculty of Chemistry, Tarbiat Moallem University, Tehran, Iran

## Abstract

In the title compound, [Hg(C_14_H_13_N_4_O_3_)Cl], the Hg^II^ atom is four-coordinated by one O atom and two N atoms from a tridentate 1-(2-eth­oxy­phen­yl)-3-(4-nitro­phen­yl)triazenide ligand and one terminal chloride ion in a distorted square-planar geometry. In the crystal structure, the mononuclear complexes are linked into pairs through C—H⋯O and C—H⋯Cl hydrogen bonds as well as π–π and C—H⋯π stacking inter­actions. In addition, weak Hg–μ^6^-arene π-inter­actions [mean distance of 3.667 (2) Å] are present between these dimers. The π–π stacking inter­actions are between aromatic rings with a centroid–centroid distance of 3.884 (2) Å. Moreover, edge-to-face inter­actions are present between eth­oxy CH groups and aromatic rings with H⋯π distances of 2.81 Å.

## Related literature

For transition-metal complexes containing 1,3-diaryltriazenide ligands, see: Moore & Robinson (1986 ▶); Vrieze & Van Koten, (1987 ▶); Horner *et al.* (2006 ▶). For related structures, see: Melardi *et al.* (2007 ▶, 2009 ▶); Rofouei *et al.* (2009 ▶).
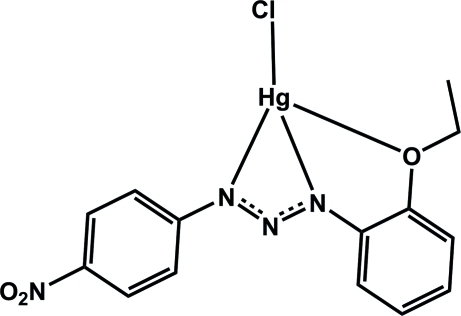


## Experimental

### 

#### Crystal data

[Hg(C_14_H_13_N_4_O_3_)Cl]
*M* *_r_* = 521.32
Monoclinic, 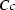

*a* = 13.4829 (5) Å
*b* = 15.5746 (6) Å
*c* = 7.7545 (3) Å
β = 107.6355 (6)°
*V* = 1551.84 (10) Å^3^
*Z* = 4
Mo *K*α radiation
μ = 10.11 mm^−1^
*T* = 120 K
0.44 × 0.10 × 0.08 mm

#### Data collection

Bruker APEXII CCD diffractometer
Absorption correction: multi-scan (*SADABS*; Bruker, 2001 ▶) *T*_min_ = 0.142, *T*_max_ = 0.618
11603 measured reflections
5612 independent reflections
5130 reflections with *I* > 2σ(*I*)
*R*_int_ = 0.031

#### Refinement

*R*[*F* ^2^ > 2σ(*F* ^2^)] = 0.022
*wR*(*F* ^2^) = 0.047
*S* = 0.76
5612 reflections
209 parameters
2 restraints
H-atom parameters constrained
Δρ_max_ = 0.87 e Å^−3^
Δρ_min_ = −0.73 e Å^−3^
Absolute structure: Flack (1983 ▶), 2739 Friedel pairs
Flack parameter: 0.003 (5)

### 

Data collection: *APEX2* (Bruker, 2005 ▶); cell refinement: *SAINT-Plus* (Bruker, 2001 ▶); data reduction: *SAINT-Plus*; program(s) used to solve structure: *SHELXS97* (Sheldrick, 2008 ▶); program(s) used to refine structure: *SHELXL97* (Sheldrick, 2008 ▶); molecular graphics: *SHELXTL* (Sheldrick, 2008 ▶); software used to prepare material for publication: *SHELXTL*.

## Supplementary Material

Crystal structure: contains datablocks I, global. DOI: 10.1107/S1600536810027819/pv2293sup1.cif

Structure factors: contains datablocks I. DOI: 10.1107/S1600536810027819/pv2293Isup2.hkl

Additional supplementary materials:  crystallographic information; 3D view; checkCIF report

## Figures and Tables

**Table 1:** Hydrogen-bond geometry (Å, °) *Cg*2 is the centroid of the C1–C6 ring.

*D*—H⋯*A*	*D*—H	H⋯*A*	*D*⋯*A*	*D*—H⋯*A*
C3—H3⋯O3^i^	0.95	2.54	3.489 (5)	174
C5—H5⋯O2^ii^	0.95	2.55	3.390 (5)	147
C9—H9⋯Cl1^iii^	0.95	2.80	3.738 (4)	169
C13—H13*A*⋯O3^iv^	0.99	2.47	3.431 (5)	162
C13—H13*B*⋯*Cg*2^v^	0.99	2.81	3.570 (4)	134

Symmetry codes: (i) 
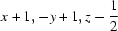
; (ii) 
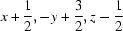
; (iii) 
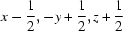
; (iv) 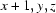; (v) 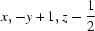.

**Fig. 1.**
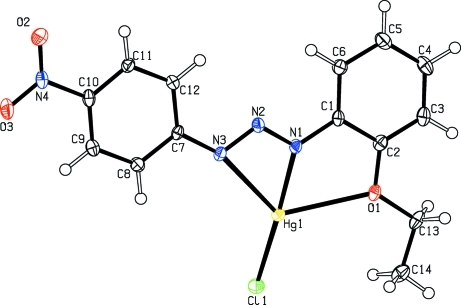
Molecular structure of the title compound, with ellipsoids drawn at 50% probability level.

**Fig. 2.**
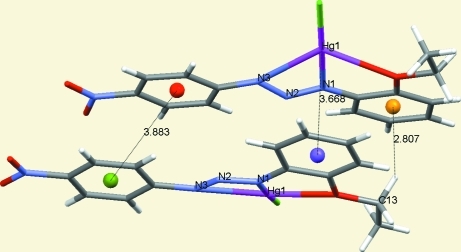
π–π, C—H···π and weak Hg-µ6-arene stacking interactions between two [Hg(C14H13N4O3)Cl] moieties.

**Fig. 3.**
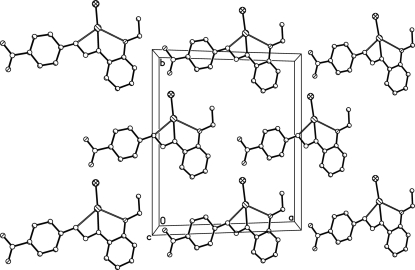
The unit cell packing diagram of the title compound along the c axis.

## supplementary crystallographic information

### Comment

Triazene compounds characterized by having a diazoamino group (–N═NN–) commonly adopt a trans configuration in the ground state. The study of transition-metal complexes containing 1,3-diaryltriaznide ligands has greatly increased in the past few years because of the versatility of their coordination forms, yielding a variety of coordination compounds with large structural diversity (Moore & Robinson, 1986;  Vrieze & Van Koten, 1987; Horner *et al.*., 2006).  The crystal structures of a few complexes related to the title compound have been reported recently (Melardi *et al.*, 2007; Rofouei *et al.*, 2009).

In the title complex (Fig. 1), the [1-(2-ethoxyphenyl)3-(4-nitrophenyl)]triazenide ion is coordinated to the central atom Hg(II) through two N atoms [Hg1—N1 = 2.070 (3) Å and Hg1—N3 = 2.711 (3) Å] and one O atom [Hg1—O1 = 2.662 (2) Å]. In addition, a Cl^-^ ion is coordinated to Hg(II) atom with the bond distance Hg1—Cl1 = 2.269 (9) Å. These bond distances agree very well with the corresponding distances reported in related structures (Melardi *et al.*, 2007; Rofouei *et al.*, 2009). The atoms of the ligand and lie in a plane (maximum deviation from coplanarity being 0.115 (4)Å for O2 while Cl1 and Hg1 lie 1.402 (3) and 0.606 (2)Å, respecively, out of this plane. The molecules of the title complex are linked to form pairs through non-classical C—H···O and C—H···Cl hydrogen bond, as well as π–π and C—H···π stacking interactions.

There are π–π stacking interactions present between aromatic rings with centroid-centroid distance of 3.884 (2) Å for *Cg*1···*Cg*1 (*Cg*1 = C7—C12, *x*, 1 - *y*, *z-*1/2), and also edge-to-face interactions are present between CH group of ethoxy with aromatic rings with H···π distance of 2.81 Å for C13—H13B···*Cg*2 (*Cg*2 = C1—C6  *x*, 1 - *y*, *z-*1/2). In addition, weak Hg-µ^6^-arene π-interactions (mean distance 3.667 (2) Å) are present between these dimers. The secondary Hg-µ^6^-arene π-interactions involve carbon atoms of the C1—C6 phenyl rings (Table 1, Fig. 2). The weak non-covalent interactions seem to play important role in the crystal packing and the formation of a desired framework. The unit cell packing of the title compound is shown in Fig. 3.

### Experimental

A methanol solution of 1-(2-ethoxyphenyl)-3-(4-nitrophenyl)triazene (0.286 g, 1 mmol) was added to a solution of mercury(II) chloride (0.270 g, 1 mmol). After mixing for 30 minutes at room temperature, solution of sodium acetate in water was added to adjust the *p*H at 6–6.2. After 2 h, a red solid was readily precipitated out. It was filtered off, washed with methanol and dried in vacuum. The orange crude material was dissolved in 10 ml of dichloromethane (CH_2_Cl_2_), and placed in a freezer without covering. After two weeks beautiful orange and air-stable crystals of the title complex were obtained by slow evaporation of the solvent; m.p. 460–462 K.

### Refinement

An absolute structure was established using Flack (1983) method. The H-atoms were placed in calculated positions with C—H = 0.95, 0.98 and 0.99 Å for aryl, methyl and methylene type H-atoms, respectively, and included in the refinement in riding mode with fixed isotropic displacement parameters (*U*_iso_(H) = 1.5U_eq_(C) for the CH_3_-groups and *U*_iso_(H) = 1.2U_eq_(C) for the other groups). The highest positive residual electron density peak of 0.87 eÅ^3^ was localized at a distance of 0.91 Å from the Hg1 atom and was meaningless.

### Crystal data

**Table:**

[Hg(C_14_H_13_N_4_O_3_)Cl]	*F*(000) = 984
*M**_r_* = 521.32	*D*_x_ = 2.231 Mg m^−^^3^
Monoclinic, *C**c*	Mo *K*α radiation, λ = 0.71073 Å
Hall symbol: C -2yc	Cell parameters from 5899 reflections
*a* = 13.4829 (5) Å	θ = 2.6–32.6°
*b* = 15.5746 (6) Å	µ = 10.11 mm^−^^1^
*c* = 7.7545 (3) Å	*T* = 120 K
β = 107.6355 (6)°	Needle, red
*V* = 1551.84 (10) Å^3^	0.44 × 0.10 × 0.08 mm
*Z* = 4	

### Data collection

**Table:**

Bruker APEXII CCD diffractometer	5612 independent reflections
Radiation source: fine-focus sealed tube	5130 reflections with *I* > 2σ(*I*)
graphite	*R*_int_ = 0.031
φ and ω scans	θ_max_ = 32.8°, θ_min_ = 2.1°
Absorption correction: multi-scan (*SADABS*; Bruker, 2001)	*h* = −20→20
*T*_min_ = 0.142, *T*_max_ = 0.618	*k* = −23→23
11603 measured reflections	*l* = −11→11

### Refinement

**Table:**

Refinement on *F*^2^	Secondary atom site location: difference Fourier map
Least-squares matrix: full	Hydrogen site location: inferred from neighbouring sites
*R*[*F*^2^ > 2σ(*F*^2^)] = 0.022	H-atom parameters constrained
*wR*(*F*^2^) = 0.047	*w* = 1/[σ^2^(*F*_o_^2^)] where *P* = (*F*_o_^2^ + 2*F*_c_^2^)/3
*S* = 0.76	(Δ/σ)_max_ = 0.002
5612 reflections	Δρ_max_ = 0.87 e Å^−^^3^
209 parameters	Δρ_min_ = −0.73 e Å^−^^3^
2 restraints	Absolute structure: Flack (1983), 2739 Friedel pairs
Primary atom site location: structure-invariant direct methods	Flack parameter: 0.003 (5)

### Special details

**Table:**

Geometry. All e.s.d.'s (except the e.s.d. in the dihedral angle between two l.s. planes) are estimated using the full covariance matrix. The cell e.s.d.'s are taken into account individually in the estimation of e.s.d.'s in distances, angles and torsion angles; correlations between e.s.d.'s in cell parameters are only used when they are defined by crystal symmetry. An approximate (isotropic) treatment of cell e.s.d.'s is used for estimating e.s.d.'s involving l.s. planes.
Refinement. Refinement of *F*^2^ against ALL reflections. The weighted *R*-factor *wR* and goodness of fit *S* are based on *F*^2^, conventional *R*-factors *R* are based on *F*, with *F* set to zero for negative *F*^2^. The threshold expression of *F*^2^ > σ(*F*^2^) is used only for calculating *R*-factors(gt) *etc*. and is not relevant to the choice of reflections for refinement. *R*-factors based on *F*^2^ are statistically about twice as large as those based on *F*, and *R*- factors based on ALL data will be even larger.

### Fractional atomic coordinates and isotropic or equivalent isotropic displacement parameters (Å^2^)

**Table:**

	*x*	*y*	*z*	*U*_iso_*/*U*_eq_	
Hg1	0.12017 (2)	0.366861 (6)	0.01531 (2)	0.01598 (3)	
Cl1	0.09959 (7)	0.22761 (6)	−0.07489 (13)	0.02390 (18)	
O1	0.29846 (19)	0.43726 (16)	0.0064 (3)	0.0186 (5)	
O2	−0.3935 (2)	0.61872 (18)	0.3415 (5)	0.0284 (6)	
O3	−0.4252 (2)	0.48304 (18)	0.3448 (4)	0.0251 (6)	
N1	0.1308 (2)	0.49607 (17)	0.0809 (4)	0.0139 (5)	
N2	0.0536 (2)	0.52465 (18)	0.1369 (4)	0.0143 (5)	
N3	−0.0072 (2)	0.46291 (18)	0.1493 (4)	0.0135 (5)	
N4	−0.3717 (2)	0.5440 (2)	0.3237 (4)	0.0178 (6)	
C1	0.1976 (2)	0.5559 (2)	0.0397 (4)	0.0141 (6)	
C2	0.2875 (3)	0.5247 (2)	0.0013 (4)	0.0158 (6)	
C3	0.3560 (3)	0.5818 (2)	−0.0381 (5)	0.0207 (7)	
H3	0.4169	0.5612	−0.0620	0.025*	
C4	0.3359 (3)	0.6695 (3)	−0.0427 (5)	0.0240 (8)	
H4	0.3840	0.7086	−0.0675	0.029*	
C5	0.2464 (3)	0.7004 (2)	−0.0112 (5)	0.0240 (7)	
H5	0.2326	0.7604	−0.0169	0.029*	
C6	0.1767 (3)	0.6436 (2)	0.0287 (5)	0.0177 (6)	
H6	0.1149	0.6646	0.0484	0.021*	
C7	−0.0947 (2)	0.4891 (2)	0.1988 (4)	0.0126 (6)	
C8	−0.1560 (3)	0.4226 (2)	0.2309 (5)	0.0162 (6)	
H8	−0.1348	0.3647	0.2252	0.019*	
C9	−0.2472 (3)	0.4396 (2)	0.2710 (5)	0.0169 (6)	
H9	−0.2888	0.3943	0.2931	0.020*	
C10	−0.2762 (3)	0.5245 (2)	0.2780 (5)	0.0158 (6)	
C11	−0.2170 (3)	0.5921 (2)	0.2465 (5)	0.0155 (6)	
H11	−0.2385	0.6498	0.2531	0.019*	
C12	−0.1261 (3)	0.5744 (2)	0.2052 (5)	0.0155 (6)	
H12	−0.0853	0.6200	0.1813	0.019*	
C13	0.3904 (3)	0.4016 (3)	−0.0273 (5)	0.0206 (7)	
H13A	0.4541	0.4228	0.0641	0.025*	
H13B	0.3930	0.4188	−0.1487	0.025*	
C14	0.3835 (3)	0.3054 (2)	−0.0161 (6)	0.0251 (8)	
H14A	0.4450	0.2792	−0.0368	0.038*	
H14B	0.3207	0.2851	−0.1083	0.038*	
H14C	0.3802	0.2891	0.1042	0.038*	

### Atomic displacement parameters (Å^2^)

**Table:**

	*U*^11^	*U*^22^	*U*^33^	*U*^12^	*U*^13^	*U*^23^
Hg1	0.01404 (5)	0.01342 (4)	0.02179 (5)	0.00069 (9)	0.00741 (3)	−0.00111 (10)
Cl1	0.0207 (4)	0.0148 (4)	0.0376 (5)	0.0004 (3)	0.0109 (4)	−0.0046 (3)
O1	0.0137 (11)	0.0211 (12)	0.0250 (13)	0.0020 (9)	0.0117 (10)	−0.0003 (10)
O2	0.0230 (14)	0.0235 (14)	0.0441 (18)	0.0042 (11)	0.0182 (13)	0.0018 (12)
O3	0.0175 (13)	0.0306 (15)	0.0307 (15)	−0.0056 (11)	0.0127 (12)	−0.0014 (12)
N1	0.0124 (12)	0.0148 (12)	0.0164 (12)	−0.0001 (10)	0.0070 (11)	−0.0015 (10)
N2	0.0123 (12)	0.0168 (13)	0.0144 (13)	−0.0011 (10)	0.0048 (10)	0.0009 (10)
N3	0.0111 (12)	0.0162 (13)	0.0133 (12)	0.0012 (10)	0.0040 (10)	0.0011 (10)
N4	0.0134 (13)	0.0239 (15)	0.0179 (14)	−0.0031 (11)	0.0074 (11)	−0.0009 (11)
C1	0.0115 (14)	0.0188 (15)	0.0127 (14)	−0.0012 (11)	0.0048 (11)	−0.0004 (12)
C2	0.0161 (15)	0.0196 (16)	0.0132 (14)	0.0017 (12)	0.0067 (12)	0.0004 (12)
C3	0.0151 (15)	0.0282 (19)	0.0209 (17)	−0.0013 (13)	0.0088 (13)	−0.0001 (14)
C4	0.0261 (19)	0.0242 (18)	0.0256 (18)	−0.0077 (15)	0.0134 (16)	0.0024 (15)
C5	0.0248 (19)	0.0191 (17)	0.0308 (19)	−0.0043 (14)	0.0123 (16)	0.0012 (15)
C6	0.0188 (16)	0.0169 (15)	0.0203 (16)	0.0005 (12)	0.0100 (13)	−0.0003 (12)
C7	0.0103 (13)	0.0176 (15)	0.0103 (13)	0.0016 (11)	0.0037 (11)	0.0007 (11)
C8	0.0168 (15)	0.0148 (14)	0.0180 (15)	−0.0013 (12)	0.0067 (12)	−0.0005 (12)
C9	0.0163 (15)	0.0197 (16)	0.0157 (15)	−0.0036 (12)	0.0065 (12)	−0.0010 (12)
C10	0.0138 (15)	0.0221 (16)	0.0135 (14)	−0.0028 (12)	0.0070 (13)	−0.0010 (12)
C11	0.0129 (14)	0.0161 (15)	0.0181 (15)	0.0012 (12)	0.0055 (12)	−0.0014 (12)
C12	0.0142 (15)	0.0144 (14)	0.0180 (15)	−0.0023 (11)	0.0053 (12)	0.0004 (12)
C13	0.0144 (15)	0.0272 (18)	0.0211 (16)	0.0060 (14)	0.0069 (13)	−0.0027 (15)
C14	0.0222 (18)	0.0262 (19)	0.0289 (19)	0.0072 (15)	0.0107 (16)	−0.0008 (15)

### Geometric parameters (Å, °)

**Table:**

Hg1—N1	2.070 (3)	C5—C6	1.392 (5)
Hg1—Cl1	2.2699 (9)	C5—H5	0.9500
Hg1—O1	2.662 (2)	C6—H6	0.9500
Hg1—N3	2.711 (3)	C7—C8	1.394 (4)
O1—C2	1.370 (4)	C7—C12	1.401 (5)
O1—C13	1.453 (4)	C8—C9	1.382 (5)
O2—N4	1.218 (4)	C8—H8	0.9500
O3—N4	1.233 (4)	C9—C10	1.385 (5)
N1—N2	1.320 (4)	C9—H9	0.9500
N1—C1	1.399 (4)	C10—C11	1.387 (5)
N2—N3	1.286 (4)	C11—C12	1.385 (5)
N3—C7	1.406 (4)	C11—H11	0.9500
N4—C10	1.467 (4)	C12—H12	0.9500
C1—C6	1.392 (4)	C13—C14	1.505 (6)
C1—C2	1.419 (4)	C13—H13A	0.9900
C2—C3	1.382 (5)	C13—H13B	0.9900
C3—C4	1.390 (6)	C14—H14A	0.9800
C3—H3	0.9500	C14—H14B	0.9800
C4—C5	1.389 (6)	C14—H14C	0.9800
C4—H4	0.9500		
			
N1—Hg1—Cl1	175.95 (8)	C1—C6—C5	120.0 (3)
N1—Hg1—O1	67.07 (9)	C1—C6—H6	120.0
Cl1—Hg1—O1	114.37 (6)	C5—C6—H6	120.0
N1—Hg1—N3	51.23 (10)	C8—C7—C12	119.8 (3)
Cl1—Hg1—N3	127.80 (6)	C8—C7—N3	115.2 (3)
O1—Hg1—N3	117.69 (8)	C12—C7—N3	124.9 (3)
C2—O1—C13	117.8 (3)	C9—C8—C7	120.9 (3)
C2—O1—Hg1	108.66 (19)	C9—C8—H8	119.5
C13—O1—Hg1	132.3 (2)	C7—C8—H8	119.5
N2—N1—C1	118.5 (3)	C8—C9—C10	118.3 (3)
N2—N1—Hg1	114.0 (2)	C8—C9—H9	120.9
C1—N1—Hg1	126.3 (2)	C10—C9—H9	120.9
N3—N2—N1	110.9 (3)	C9—C10—C11	122.2 (3)
N2—N3—C7	114.2 (3)	C9—C10—N4	119.1 (3)
N2—N3—Hg1	83.78 (18)	C11—C10—N4	118.7 (3)
C7—N3—Hg1	161.0 (2)	C12—C11—C10	119.1 (3)
O2—N4—O3	123.4 (3)	C12—C11—H11	120.4
O2—N4—C10	119.0 (3)	C10—C11—H11	120.4
O3—N4—C10	117.6 (3)	C11—C12—C7	119.7 (3)
C6—C1—N1	122.4 (3)	C11—C12—H12	120.2
C6—C1—C2	119.5 (3)	C7—C12—H12	120.2
N1—C1—C2	118.1 (3)	O1—C13—C14	107.4 (3)
O1—C2—C3	124.9 (3)	O1—C13—H13A	110.2
O1—C2—C1	115.2 (3)	C14—C13—H13A	110.2
C3—C2—C1	119.8 (3)	O1—C13—H13B	110.2
C2—C3—C4	120.0 (3)	C14—C13—H13B	110.2
C2—C3—H3	120.0	H13A—C13—H13B	108.5
C4—C3—H3	120.0	C13—C14—H14A	109.5
C5—C4—C3	120.7 (3)	C13—C14—H14B	109.5
C5—C4—H4	119.7	H14A—C14—H14B	109.5
C3—C4—H4	119.7	C13—C14—H14C	109.5
C4—C5—C6	119.9 (4)	H14A—C14—H14C	109.5
C4—C5—H5	120.0	H14B—C14—H14C	109.5
C6—C5—H5	120.0		
			
N1—Hg1—O1—C2	−16.5 (2)	N1—C1—C2—O1	0.9 (4)
Cl1—Hg1—O1—C2	159.32 (18)	C6—C1—C2—C3	3.1 (5)
N3—Hg1—O1—C2	−24.7 (2)	N1—C1—C2—C3	−179.3 (3)
N1—Hg1—O1—C13	176.7 (3)	O1—C2—C3—C4	178.8 (3)
Cl1—Hg1—O1—C13	−7.4 (3)	C1—C2—C3—C4	−1.0 (5)
N3—Hg1—O1—C13	168.5 (3)	C2—C3—C4—C5	−1.2 (5)
O1—Hg1—N1—N2	−173.2 (2)	C3—C4—C5—C6	1.2 (6)
N3—Hg1—N1—N2	−2.54 (18)	N1—C1—C6—C5	179.4 (3)
O1—Hg1—N1—C1	19.7 (2)	C2—C1—C6—C5	−3.1 (5)
N3—Hg1—N1—C1	−169.6 (3)	C4—C5—C6—C1	0.9 (6)
C1—N1—N2—N3	172.7 (3)	N2—N3—C7—C8	−173.8 (3)
Hg1—N1—N2—N3	4.5 (3)	N2—N3—C7—C12	10.7 (4)
N1—N2—N3—C7	−176.7 (3)	C12—C7—C8—C9	−0.7 (5)
N1—N2—N3—Hg1	−3.1 (2)	N3—C7—C8—C9	−176.5 (3)
N1—Hg1—N3—N2	2.39 (17)	C7—C8—C9—C10	0.1 (5)
Cl1—Hg1—N3—N2	−172.60 (15)	C8—C9—C10—C11	0.0 (5)
O1—Hg1—N3—N2	12.1 (2)	C8—C9—C10—N4	−178.9 (3)
N1—Hg1—N3—C7	164.2 (7)	O2—N4—C10—C9	175.2 (3)
Cl1—Hg1—N3—C7	−10.8 (7)	O3—N4—C10—C9	−3.7 (5)
O1—Hg1—N3—C7	173.8 (6)	O2—N4—C10—C11	−3.6 (5)
N2—N1—C1—C6	−10.1 (5)	O3—N4—C10—C11	177.4 (3)
Hg1—N1—C1—C6	156.5 (3)	C9—C10—C11—C12	0.5 (5)
N2—N1—C1—C2	172.4 (3)	N4—C10—C11—C12	179.3 (3)
Hg1—N1—C1—C2	−21.0 (4)	C10—C11—C12—C7	−1.0 (5)
C13—O1—C2—C3	1.7 (5)	C8—C7—C12—C11	1.1 (5)
Hg1—O1—C2—C3	−167.2 (3)	N3—C7—C12—C11	176.5 (3)
C13—O1—C2—C1	−178.5 (3)	C2—O1—C13—C14	−179.4 (3)
Hg1—O1—C2—C1	12.6 (3)	Hg1—O1—C13—C14	−13.6 (4)
C6—C1—C2—O1	−176.7 (3)		

### Hydrogen-bond geometry (Å, °)

**Table:**

Cg2 is the centroid of the C1–C6 ring.

**Table:**

*D*—H···*A*	*D*—H	H···*A*	*D*···*A*	*D*—H···*A*
C3—H3···O3^i^	0.95	2.54	3.489 (5)	174
C5—H5···O2^ii^	0.95	2.55	3.390 (5)	147
C9—H9···Cl1^iii^	0.95	2.80	3.738 (4)	169
C13—H13A···O3^iv^	0.99	2.47	3.431 (5)	162
C13—H13B···Cg2^v^	0.99	2.81	3.570 (4)	134

Symmetry codes: (i) *x*+1, −*y*+1, *z*−1/2; (ii) *x*+1/2, −*y*+3/2, *z*−1/2; (iii) *x*−1/2, −*y*+1/2, *z*+1/2; (iv) *x*+1, *y*, *z*; (v) *x*, −*y*+1, *z*−1/2.
